# Investigating knockdown resistance (*kdr*) mechanism against pyrethroids/DDT in the malaria vector *Anopheles funestus* across Africa

**DOI:** 10.1186/s12863-017-0539-x

**Published:** 2017-08-09

**Authors:** Helen Irving, Charles S. Wondji

**Affiliations:** 1 Vector Biology Department, Liverpool School of Tropical Medicine, Pembroke Place, Liverpool, L3 5QA UK; 2 LSTM research Unit at the Organisation de Coordination pour la lutte contre les Endemies en Afrique Centrale, P.O Box 288, Yaoundé, Cameroon

**Keywords:** Mosquito, Malaria, *Anopheles Funestus*, Insecticide resistance, Sodium channel gene, Knockdown resistance

## Abstract

**Background:**

Understanding the molecular basis of insecticide resistance is key to improve the surveillance and monitoring of malaria vector populations under control. In the major malaria vector *Anopheles funestus*, little is currently known about the role of the knockdown resistance (*kdr*) mechanism. Here, we investigated the presence and contribution of knockdown resistance (*kdr*) to pyrethroids/DDT resistance observed in *Anopheles funestus* across Africa.

**Results:**

Pyrosequencing genotyping and sequencing of the voltage gated sodium channel (VGSC) gene did not detect the common L1014F mutation in field collected *An. funestus* across Africa. Amplification and cloning of the full-length of the sodium channel gene in pyrethroid resistant mosquitoes revealed evidences of alternative splicing events with three transcripts of 2092, 2061 and 2117 amino acids (93% average similarity to *An. gambiae*). Several amino acid changes were detected close to the domain II of the protein such as L928R, F938 W, I939S, L802S and T1008 M. However, all these mutations are found at low frequency and their role in pyrethroid resistance could not be established. The presence of the exclusive alternative splicing at exon 19 was not associated with resistance phenotype. Analysis of patterns of genetic diversity of the VGSC gene revealed a high polymorphism level of this gene across Africa with no evidence of directional selection suggesting a limited role for knockdown resistance in pyrethroid resistance in *An. funestus*. Patterns of genetic differentiation correlate with previous observations of the existence of barriers to gene flow Africa-wide with southern population significantly differentiated from other regions.

**Conclusion:**

Despite an apparent limited role of knockdown resistance in *An. funestus*, it is necessary to continue to monitor the contribution of the mutations detected here as increasing selection from insecticide-based interventions may change the dynamic in field populations as previously observed in other vectors.

**Electronic supplementary material:**

The online version of this article (doi:10.1186/s12863-017-0539-x) contains supplementary material, which is available to authorized users.

## Background

Malaria prevention relies heavily on insecticide-based vector control interventions such as Long Lasting Insecticide Nets (LLINs) and Indoor Residual Spraying (IRS) [1]. However increasing reports of insecticide resistance is a serious cause of concern for the long-term effectiveness of these control tools. In the major malaria vector *An. funestus s.s.*, resistance to pyrethroid insecticides (only class recommended for bed net impregnation) is spreading across Africa with reports of resistance in all regions of the continent. Resistance to both type I and II pyrethroids firstly detected in southern Africa [2, 3] has since spread across the region [4–9]. Similar reports of resistance have been made in East Africa [10–12], in West Africa [13–17] and Central Africa [18, 19]. To ensure a continued effectiveness of the current control tools it is imperative to implement successful resistance management strategies. However, this requires a thorough understanding of the molecular basis of resistance and also the design of field applicable diagnostic tools to easily detect and monitor the spread of resistance in the field.

To date, investigations of the underlying molecular basis of all these reports of pyrethroid resistance have indicated that metabolic resistance through over-expression of cytochrome P450 genes is the major resistance mechanism in *An. funestus s.s.* [20–24]. Synergist assay with Piperonyl Butoxide (PBO), a P450 inhibitor, have consistently showed a near full recovery of susceptibility to pyrethroids when mosquitoes were pre-exposure to PBO [2, 12] suggesting a major role played by cytochrome P450s. This was further supported by in-depth genetic and molecular characterisation of metabolic resistance in both laboratory and field population of *An. funestus s.s.* in southern Africa which revealed that the duplicated cytochrome P450 genes, *CYP6P9a* and *CYP6P9b* beside *CYP6M7* are playing key role in pyrethroid resistance [21, 23, 25–27].

No knockdown resistance (*kdr*) mutation has been detected in *An. funestus s.s.* in stark contrast to other mosquito species including the major malaria vector *An. gambiae* for which *kdr* mutations, notably at the 1014 codon, have been widely reported [28–30]. Indeed, despite sequencing of the Exon 20 fragment, no *kdr* mutation has been detected in *An. funestus* [8, 12–14] although some amino acid changes (I877L, V881 L and A1007S in Cameroon, F1021C in Uganda) have been recently reported but with no association with resistance [12, 18]. However, a comparative analysis of the haplotype diversity of the voltage-gated sodium channel (VGSC) gene between permethrin resistant and susceptible mosquitoes from Malawi showed a possible correlation between resistance phenotype and haplotype distribution, suggesting that other *kdr* mutations different to the classical L1014F/S mutations could be associated with pyrethroid resistance in *An. funestus s.s.* [8, 9]. Indeed, in other insect species, different *kdr* resistance mutations than the L1014F have been detected such as in *Aedes aegypti* with mutations in 1011, 1016 and 1534 codon positions [31, 32] or in *Musca domestica* with 918 mutation [33] or recently in *An. gambiae* with the N1575Y mutation [34]. It cannot be excluded that this could be the case in *An. funestus s.s.*.

It is known that some resistance alleles could occur at a low frequency in field populations and gradually increased for many years without been detected, and then when a tipping point is reached, the frequency of that allele could significantly increase in the population in a short period of time. This phenomenon was observed in *Ae. aegypti* in Mexico [1] and also in *An. coluzzii* (previously *An. gambiae* M form) for which very low frequencies of the 1014F *kdr* allele (<2%) were detected before 2000 in Vallée du Kou in Burkina Faso [35] before a significant increase to 75% in ten years [36]. Because it is important to detect resistance allele when it still at low frequency in vector populations in order to facilitate the implementation of suitable resistance management strategies, it is necessary to keep on monitoring for the presence of *kdr* mutations in *An. funestus* across the continent. The sequencing of the full-length of the VGSC gene will help to identify such resistance mutation and could lead to the design of suitable diagnostic tool to detect and monitor the evolution of this resistance in natural populations of this vector.

The present study aimed at establishing whether beside the predominant metabolic resistance mechanism, the target site resistance through knockdown resistance was also playing a role in pyrethroid resistance in *An. funestus s.s.*. To elucidate this question we cloned and sequenced the full length 6 kb of the VGSC gene in several populations of *An. funestus* across the continent, detected potential *kdr* resistance mutations and assessed their correlation with pyrethroid or DDT resistance. Furthermore, we assessed whether possible selection was acting on this gene and established pattern of genetic structure between African *An. funestus s.s.* populations based on VGSC diversity profiles.

## Methods

### Mosquito collection

Blood-fed female *An. funestus* adults resting indoor were collected in houses between 06.00 and 12.00 AM in several countries as previously reported. These locations include Mbinga (Katete District) (14° 11′ 0″ S, 31° 52′ 0″ E) in eastern Zambia in October 2010 [27]; Chikwawa District (0° 45′ N, 34° 5′E) in southern Malawi in July 2009 and April 2010 [23]; Tihuquine (Chokwe District) (24° 33′ 37″ S, 33° 1′ 20″ E) in southern Mozambique in January 2009 (Cuamba et al. 2010); Tororo in eastern Uganda (0°41′N, 34°10′E) [11]; Pahou (6° 23′ N, 2° 13′E) in southern Benin [13]. The collection method and the rearing were done as described previously [11]. Briefly, F_1_ adults were generated from field collected female mosquitoes using the forced-egg laying method [11] and were randomly mixed in cages for subsequent experiments.

### PCR-species identification

All females used for individual oviposition were morphologically identified as belonging to the *An. funestus* group according to the key of Gillies and Coetzee [37]. Genomic DNA was extracted using the Livak protocol [38]. A PCR was carried out using the protocol of Koekemoer et al. [39] to confirm that all females that laid eggs were *An. funestus s.s..*

### Genotyping of 1014 *kdr* coding position in field *An. funestus*

A pyrosequencing assay was used to genotype the *VGSC* 1014 coding position in a set of 100 field collected adult female *An. funestus* from southern Africa (Zambia, Mozambique, Malawi), from West Africa (Benin) and in East Africa (Uganda) to check the possible presence of the L1014F *kdr* mutation in this species. The pyrosequencing reactions were carried out according to Wondji et al. [40, 41] with a design allowing the simultaneous detection of both possible L1014F and L1014S *kdr* mutations reported in *An. gambiae*. Details of the pyrosequencing assay and the primers used (Additional file 1: Table S1) have been previously published [13].

### Sequence analysis of exon19 in Uganda mosquitoes

A genomic fragment spanning exon 19 and the intron between exon 19 and 20 was amplified and directly sequenced for 9 resistant and 4 permethrin susceptible mosquitoes from Tororo. The genomic fragment was amplified using primers KdrEx19F and KdrEx19R (Additional file 1: Table S2). The PCR was carried out using 10 pmol of each primers and 30 ng of genomic DNA as template in 25 μl reactions containing 1X Kapa Taq buffer, 0.2 mM dNTPs, 1.5 mM MgCl_2_, 1 U Kapa Taq (Kapa Biosystems, Wilmington, MA, USA). The cycle parameters were: 1 cycle at 95 °C for 5 min; 35 cycles of 94 °C for 30s, 57 °C for 30s and elongation at 72 °C for 1 min; followed by 1 cycle at 72 °C for 10 min. Sequences were aligned using ClustalW [42] while haplotypes reconstruction and polymorphism analysis were done using DnaSP v5.10 [43]. The phylogenetic Neighbour-joining trees were constructed using MEGA 6.0 [44].

### cDNA preparation and full length amplification of voltage-gated sodium channel gene (VGSC)

RNA was extracted from three batches of ten 2- to 5-day-old *An. funestus* females alive after exposure to 0.75% permethrin (Resistant, R) and unexposed mosquitoes samples from Malawi, Mozambique, Zambia and Benin and from the susceptible FANG strain (Susceptible, S) using the Picopure RNA Isolation Kit (Thermo Fisher Scientific, Waltham, MA, USA). The quantity and quality of the extracted RNA were assessed using a NanoDrop ND1000 spectrophotometer (Thermo Fisher Scientific, Waltham, MA, USA) and Bioanalyzer (Agilent, Santa Clara, CA, USA), respectively. 1 μg of total RNA from each of the three biological replicates from the resistant populations (R) and FANG (S) was used as the template for cDNA synthesis using Superscript III (Invitrogen, Carlsbad, CA, USA) with oligo-dT20 and RNase H according to the manufacturer’s instructions.

The full length of the Voltage Gated Sodium Channel gene (VGSC) was amplified using cDNA from permethrin resistant mosquito samples from Malawi, Mozambique, Zambia and Benin and from the susceptible FANG strain. Amplification was performed using the Phusion High-Fidelity DNA Polymerase (Thermo Fisher Scientific, Waltham, MA, USA) in a 25 μl reaction containing 10 pmol of each primers, 30 ng of cDNA as template, 1X Phusion® HF Buffer, 0.2 mM dNTPs, 1.5 mM MgCl_2_, 1 U Phusion Polymerase. The PCR conditions were as follows: 1 cycle at 95 °C for 5 min; 35 cycles of 94 °C for 20s, 57 °C for 30s and elongation at 72 °C for 2 min 30s; and 1 cycle at 72 °C for 5 min. The primers used are listed in Additional file 1: Table S1. The PCR products were purified using the QIAquick PCR Purification Kit (Qiagen, Valencia, CA, USA) and directly sequenced on both strands using a set of primers listed in Additional file 1: Table S1. To confirm mutations obtained after direct sequencing and to resolve the issue of overlapping sequencing peaks due to the presence of alternative splicing positions a portion of VGSC from exon 1 to exon 20 was cloned into the pJET1.2/blunt cloning vector using the CloneJET™ PCR Cloning Kit (Thermo Fisher Scientific, Waltham, MA, USA). Positive clones were purified by QIAprep® Miniprep (Qiagen, Valencia, CA, USA) and sequenced on both strands using a set of primers listed in Additional file 1: Table S2. Polymorphic positions were detected through manual analysis of sequence traces using BioEdit and as sequence differences in multiple alignments using ClustalW [42].

### Analysis of alternative splicing of exon19 with intron amplification

An attempt was made to see if there was any association (difference in frequency) between the distribution of alternative splice version of exon 19 and susceptibility to pyrethroid as the FANG mosquitoes tended to only have the 19d form while the 19c and d were both present in resistant field samples. To assess this, a portion of genomic DNA spanning the exon 19 to 20 including the intron was cloned and sequenced using primers Exon18kdrF and KdrEx20R (Additional file 1: Table S2) between resistant and susceptible mosquitoes from Malawi, Benin and Uganda.

### Pyrosequencing of candidate *kdr* mutations

Pyrosequencing assays were designed to genotype field populations for the candidate *kdr* mutations detected in respective countries in order to assess their possible involvement in pyrethroid resistance in Uganda, Malawi, Mozambique and Zambia. The pyrosequencing reactions were performed as previously described [36, 41, 45]. Briefly, three sequence specific primers (Additional file 1 Table S1) designed for each mutation using the software provided by Pyrosequencing AB (http://www.pyrosequencing.com) were used for amplification and genotyping of each mutation. Target DNA fragments for each mutation were first PCR-amplified in a reaction containing 10 pmol each of forward and biotinylated reverse primer with the same conditions as described previously [46].

### Genetic differentiation of *An. funestus* across Africa based on a portion of the VGSC gene

A portion of the VGSC spanning intron 19 and exon 20 has previously been sequenced in Uganda, Kenya, Benin and Malawi in separate studies [8, 12, 13]. These sequences were used in the present study to assess the patterns of genetic differentiation of *An. funestus* across Africa based on the *VGSC* gene by estimating the levels of pair-wise genetic differentiation between these populations. Analyses were performed as implemented in dnaSP 5.10 using the *K*_*ST*_ statistic [47]. The significance of the *K*_*ST*_ estimates was assessed by permutation of subpopulation identities and re-calculating *K*_*ST*_ 10,000 times.

#### Phylogenetic tree of VGSC haplotypes

A maximum likelihood phylogenetic tree was constructed for the VGSC haplotypes from Uganda, Kenyan, Benin and Malawi samples using MEGA 6.0 [44]. The best-fit substitution model was firstly assessed based on the Bayesian Information Criterion (BIC) and used to generate the maximum likelihood tree as implemented in MEGA 6.0 [44] with 500 bootstrap replications to assess the robustness of the tree. A phylogenetic tree was also built with pairwise *N*_*ST*_ genetic distances between samples using MEGA 6.0.

## Results

### Pyrosequencing genotyping of L1014 across Africa

The pyrosequencing assay was successfully used to genotype the *kdr* 1014 coding position in a set of 100 field collected female adults *An. funestus* from southern Africa (Zambia, Mozambique, Malawi), from West Africa (Benin). Only the TTA codon for Leucine (L1014) (Additional file 2: Figure S1A) was detected indicating the absence of the 1014F or 1014S *kdr* mutations common in *An. gambiae*. However, in Uganda, two field mosquitoes were found to be heterozygotes TTA/T (Additional file 2: Figure S1B) suggesting the possible presence of the 1014F mutation in the *An. funestus* population of this country. Further steps were taken to validate this heterozygote status by amplifying a portion of exon 20 spanning the 1014 codon in these two mosquitoes. However, analysis of the sequencing traces confirm the absence of the 1014F mutation as both mosquitoes were TTA homozygotes. In addition, pyrosequencing genotyping of the 1014 position was further performed in 80 F_1_ mosquitoes which was permethrin resistant and 80 susceptible individuals from Tororo in Uganda; but no mutation was detected. Therefore, the two heterozygote TTA/T could be the result of PCR contamination from *An. gambiae* or induced by pyrosequencing errors.

### Sequence analysis of exon19 in Uganda mosquitoes

**Fig. 1:**
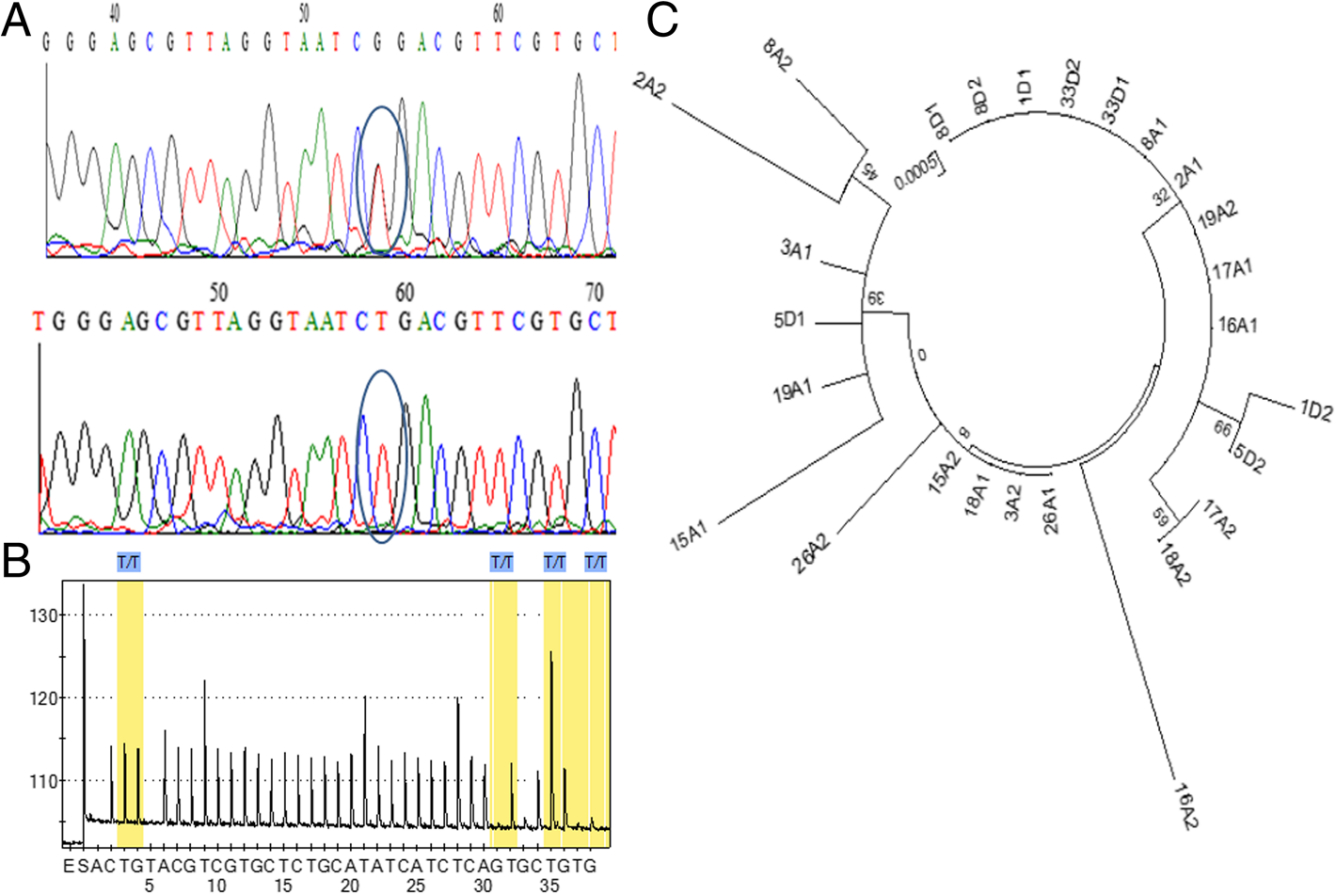
Polymorphism patterns in Exon 19 of the VGSC gene. **a** Detection of a CTG (*top*) to CGG (*bottom*) mutation inducing a L-to-R (arginine) amino acid change at codon 928 located in the IIS5 transmembrane segment. **b** Pyrosequencing chromatogram of L928R genotyping showing the CTG allele (**c**) Maximum likelihood phylogenetic tree of the 841 bp fragment spanning exon 19 showing a lack of correlation between haplotypes and resistance phenotype. Samples with A are those alive (resistant) after 1 h exposure to permethrin whereas those with D, are those dead (susceptible)

Because *kdr* mutation could occur in other exons such as exon 19 where *kdr* mutations have previously been detected in other species [48], the Ugandan population was also assessed for the presence of polymorphism possibly associated with permethrin resistance in exon 19. Nine permethrin resistant and four susceptible mosquitoes from Tororo in Uganda were successfully sequenced for an 841 bp portion of the VGSC gene spanning the Exon 19 and part of intron 19. In total 24 substitution sites were detected with 13 haplotypes. Only 3 substitutions were detected on a 101 bp portion of exon19 with one, detected in a single resistant mosquito, leading to an amino acid change with the replacement of a leucine in codon 928 by an arginine (L928R) (Fig. 1a). Additionally, significant estimates (*P* < 0.05) of Tajima D (−1.96) and Fu and Li D’ (−2.89) were observed for the total sample indicating possible signature of positive selection on this fragment of VGSC in Tororo. However, the pyrosequencing genotyping of the L928R mutation in 50 permethrin resistant and 50 susceptible only successfully amplified the wild CTG genotype for L928 (Fig. 1b). Furthermore, construction of the maximum likelihood tree of the haplotypes did not detect a pattern of haplotype clustering according to phenotype (Fig. 1c).

### Sequencing of full-length VGSC gene to detect potential *kdr* mutations

The detection of the L928R mutations and the previously reported association between VGSC haplotypes and pyrethroid resistance in Malawi [8] suggested that other potential *kdr* mutation could be identified in other exons of the VGSC gene. Therefore, a full-length cDNA of this gene was cloned and sequenced in field populations from Mozambique, Uganda and Benin. The full length of VGSC was successfully amplified in *An. funestus* with a size of 6276 bp corresponding to 2092 amino acids in a Mozambique clone (Acc No. KY499806), 6183 bp and 2061 amino acids in a clone from Uganda (Acc No. KY499807) and 6351 bp and 2117 amino acids in a clone from Benin (Acc No. KY499805). These complete sequences were compared to that of the FUMOZ strain obtained from the recently sequenced genome of *An. funestus* (AFUN000494-RA) of 6360 bp and 2120 amino acids obtained from VectorBase. These deduced amino acid sequences of *An. funestus* share 93% similarity with that of *An. gambiae* (AGAP004707-RA), 94% with that of *An. minimus* (AMIN007480-RA) and 91% with that of *Aedes aegypti* (AAEL006019-RA).

**Fig. 2:**
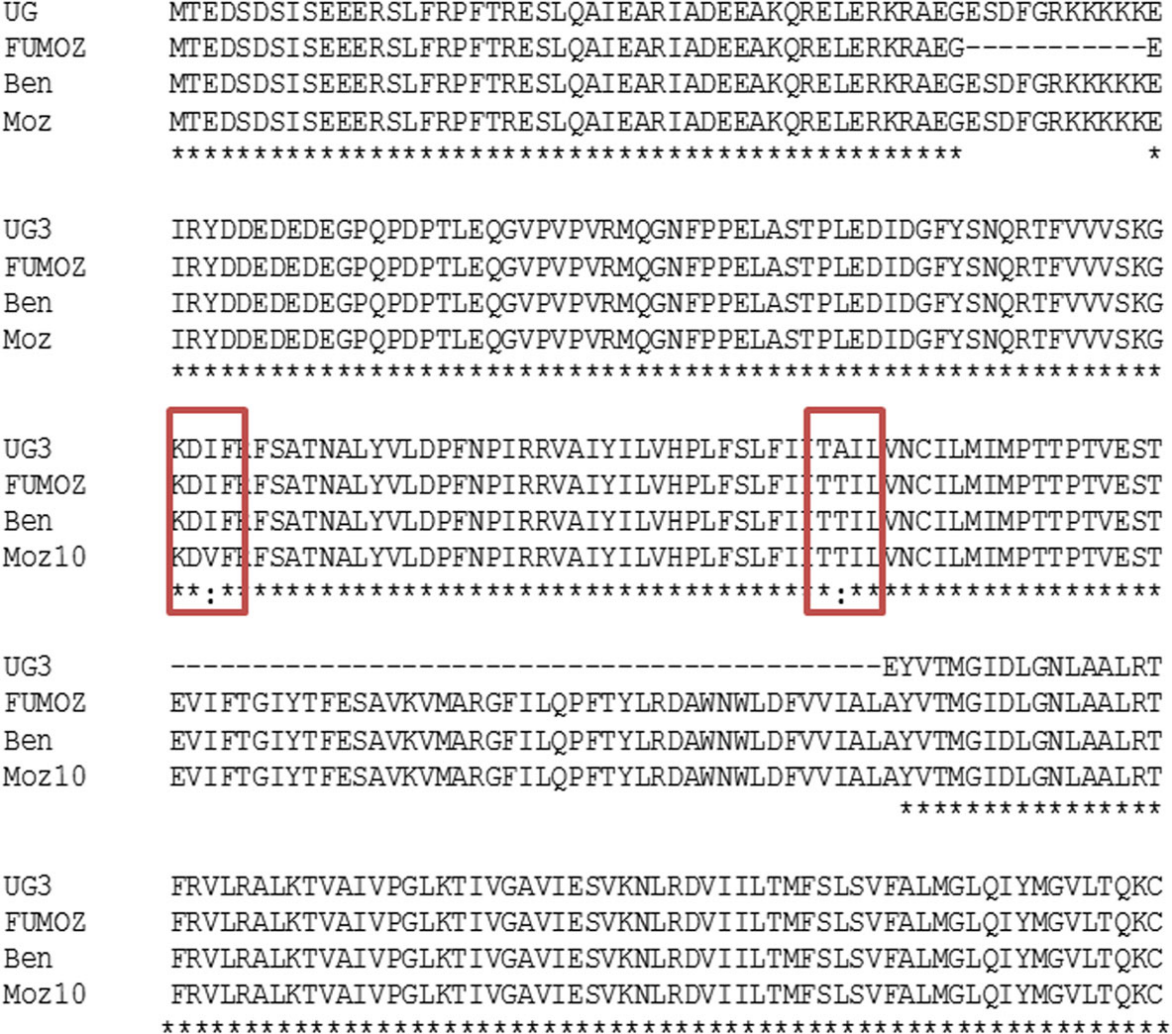
Alignment of the amino acid sequence of Exon 1 of the Voltage-Gated sodium channel gene. The polymorphic positions are shown including the two amino acid replacements I123V mutation in Mozambique (MOZ) and T161A mutation in Uganda (UG) as well as optional splice regions such as the 43 amino acid indel observed in Uganda (UG) and also reported in *An. gambiae* (Davies et al. 2007). Ben is for Benin, Moz for Mozambique and UG for Uganda

Alignment of the four *An. funestus* VGSC amino acid sequences revealed several mutations (Fig. 2) probably a result of alternative splicing events in this gene as previously reported in *An. gambiae* (Davies et al. 2007). These are characterised by insertion or deletion of portion of the gene such as the 33 bp in exon 2 detected in Mozambique encoding the “SDFGRKKKKKE” amino acid stretch. In exon 5, a 129 bp encoding domain I (S2-S3) is observed in the cDNA from Uganda (Fig. 2). Additionally, six amino acid changes were also observed with the majority being replacement between amino acids of same classes, such as I123V mutation in Mozambique. However, some amino acid changes such as the P625S (ANLG P/S RHSSY) observed only in FUMOZ could impact the activity of the protein.

**Fig. 3:**
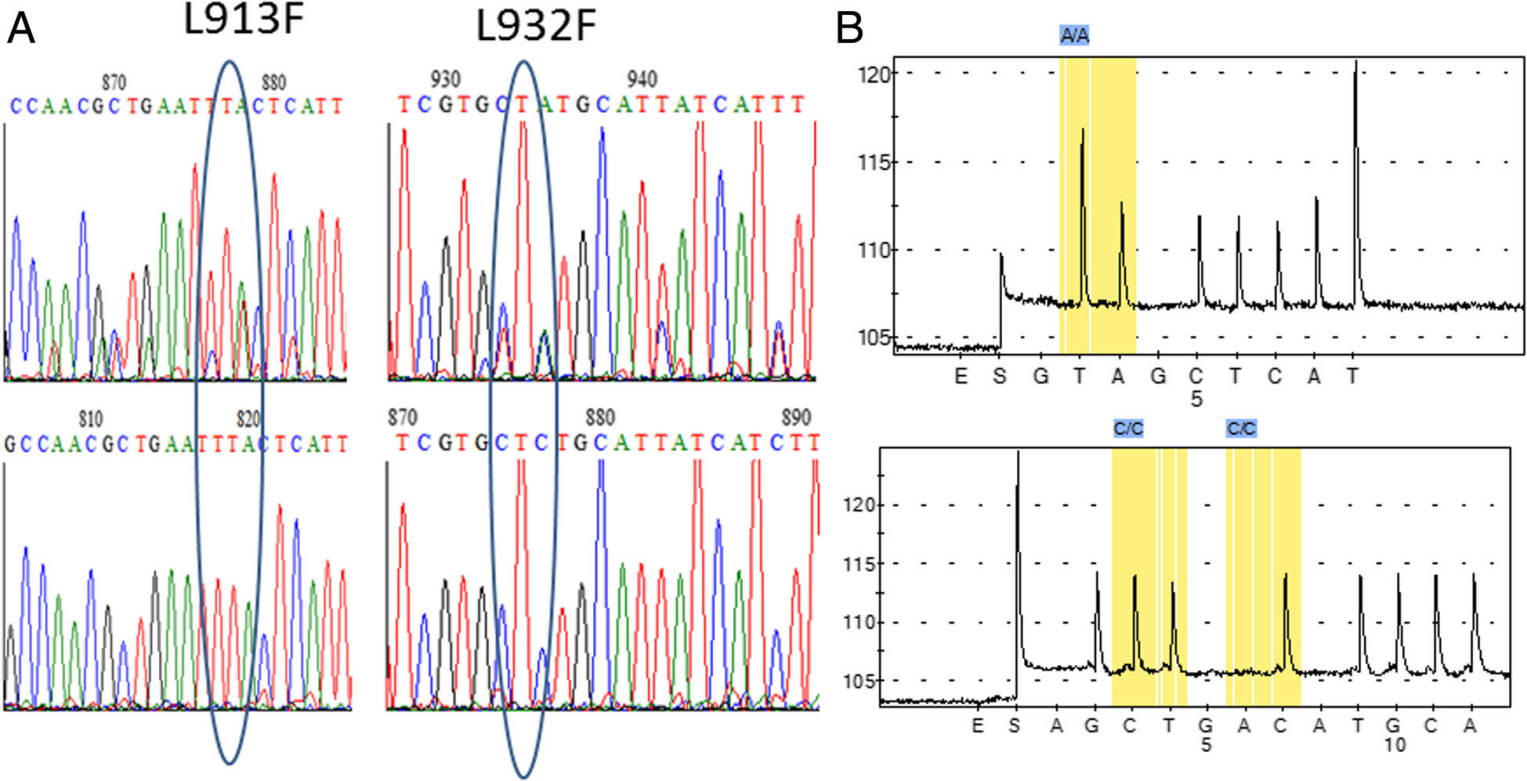
Alternative splicing on Exon 19: (**a**) Sequencing chromatograms showing (*top*) overlapping peaks from the presence of exon19c and exon19d (Davies et al. 2007) only in field pyrethroid resistant populations whereas no alternative splicing is observed for the FANG laboratory susceptible strain (*bottom*) suggesting an association between alternative splicing and resistance phenotype. Two potential mutations could lead to a L913F close to the I915M super *kdr* and also the L932F mutation shown to confer pyrethroid resistance in *Culex quinquefasciatus* (Davies et al. 2007). **b** Pyrosequencing chromatograms showing that only the TTA (L913) and CTC (L932) genotypes were detected across Africa

Furthermore, to detect potential mutations associated with pyrethroid resistance, pools of cDNA from permethrin resistant mosquitoes from Mozambique, Malawi, Zambia, Benin, Uganda and from the susceptible FANG laboratory strain were directly sequenced. Analysis of these sequences confirmed the absence of the 1014 mutation in *An. funestus*. A main variation was observed at exon 19 with several polymorphisms observed in all samples but not in the susceptible FANG (Fig. 3a) reflecting the presence of both mutually exclusive exon 19c and 19d as previously reported in *Culex quinquefasciatus* [48]. The FANG pooled cDNA sequence was equivalent to exon 19d while the field resistant samples exhibited both exon 19c and 19d. Among the many overlapping peaks were the possibility of two potential mutations which could lead to a L913F close to the I915M super *kdr* and also the L932F mutation (Fig. 3a) shown to confer pyrethroid resistance in *Culex quinquefasciatus* (Davies et al. 2007).

**Fig. 4:**
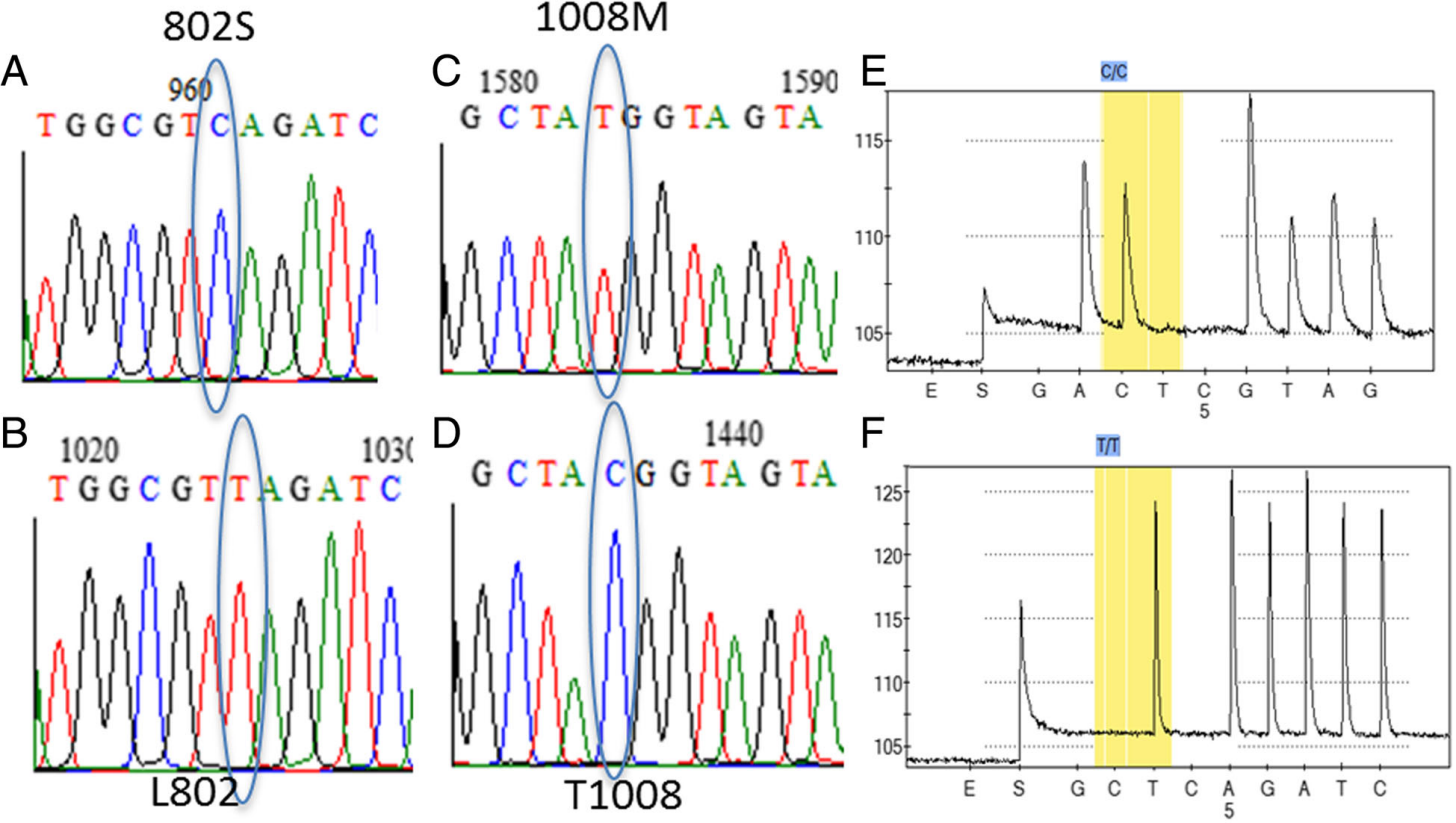
Detection of potential *kdr* mutations in the domain II of VGSC gene after cloning. **a** Sequencing chromatogram showing the TCA genotype for resistant 802S allele whereas (**b**) is for the L802 wild allele. **c** Sequencing chromatogram showing the TGG genotype for resistant 1008 M allele whereas (**d**) is for the T1008 wild allele. **e** Pyrosequencing chromatogram showing the TTA (L802) genotype whereas (**f**) is for the TGG (T1008) genotype

Because potential resistance mutations are reported in exon 19c and d in *Culex quinquefasciatus* [48], further sequencing of 26 individual clones of the fragment spanning exon 1 to 20 was carried out in Mozambique, Malawi, Zambia and Benin. This revealed a predominance of exon 19c (21 out of 26) over exon 19d (5 out of 26). The L913F and L932F mutations were not observed from the cloned samples and this was further validated by the pyrosequencing genotyping in 50 permethrin resistant and 50 susceptible individuals from Malawi and Zambia where only the wild type alleles were detected (Fig. 3b). Additionally, none of the other mutations observed in *Cx quinquefasciatus* (I915M in 19c and I936V in 19d) was detected. However, two amino acid changes were detected in Mozambique samples (each in a single clone), a T-to-C mutation at codon 802 leading to L802S substitution (TTA to TCA) in exon 18 (Fig. 4a and b) and a C-to-T at codon 1008 leading to T1008 M replacement (ACG to ATG) at exon 20 (Fig. 4c and d) which is located at the vicinity of the common L1014F mutation [LAT (1008) VVIGNL (1014) V]. Again, the pyrosequencing genotyping in 50 permethrin resistant and 50 susceptible from Malawi and Mozambique only detected the wild type alleles (Fig. 4e and f) suggesting that these mutations are probably just present at a very low frequency in the population and play no or only very minor role in the observed pyrethroid resistance The L802S and T1008 M mutation were also not found in the FUMOZ strain. One additional amino acid mutation, I818N, was also detected in Benin in a single clone but correlation with resistance was not assessed.

### Analysis of alternative splicing of exon19 with intron amplification

Attempt was made to see if there was any association (difference in frequency) between the distribution of alternative splice version of exon 19 and susceptibility to pyrethroid as the FANG mosquitoes tended to only have one form while the 19c and d were both present in field resistant samples. To assess this, a portion of genomic DNA spanning the exon 18 to 20 including the intron was cloned and sequenced between resistant and susceptible mosquitoes from Malawi, Benin and Uganda. However, cloning of a genomic portion of exon18 to exon 20 between individual permethrin resistant and susceptible mosquitoes revealed that both alternative splice exons were equally present in both phenotypes with apparently no association with pyrethroid resistance.

### Population structure of VGSC gene across African populations of *An. funestus*

Samples from 4 countries were used to assess patterns of genetic variability of the VGSC gene across African populations of *An. funestus*. These countries are Benin in West Africa, Uganda and Kenya in East Africa and Malawi in Southern Africa.

#### Haplotype distribution of VGSC across Africa

**Fig. 5:**
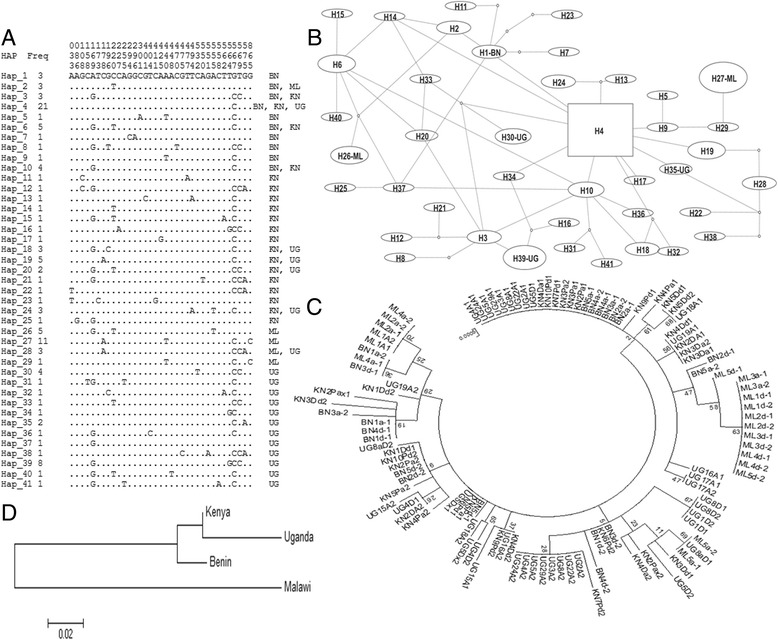
Africa-wide diversity pattern of fragment of VGSC spanning exon 20. **a** Haplotype diversity patterns of the 875 bp fragment in Benin (BN, West Africa), Uganda (UG, East Africa), Kenya (KN, East Africa) and Malawi (ML, southern Africa). **b** TCS haplotype network showing a high polymorphism of the VGSC fragment with high number of mutational steps between haplotypes. The predominant haplotype H4 is completely absent in southern Africa further suggesting barriers to gene flow between this region and others. **c** Maximum likelihood phylogenetic tree of the 875 bp fragment spanning exon 20 that Malawi haplotypes always cluster separately from those of other regions. **d** Neighbor-joining tree of the genetic distances showing that Malawi is genetically differentiated from the other regions

A total of 41 haplotypes were observed across Africa for the 875 bp fragment of VGSC spanning intron 19 and exon 20 with the lowest haplotype diversity observed in Malawi (Table 1) where a possible association between VGSC diversity and pyrethroid resistance has previously been observed [8]. Overall, the VGSC exhibits a high polymorphism level for all the countries except in Malawi. This high polymorphism is shown first by the fact that a majority of haplotypes occurred as singletons (25/41), although a major haplotype (H4; 21/110) (Fig. 5a) is observed although only shared by the West and East African countries but not by Malawi. Second, the high number of mutational steps between haplotypes as shown on the TCS haplotype network further highlights the high polymorphism of the VGSC gene in these populations (Fig. 5b). Thirdly, there is no indication of selection acting on these populations as shown by the values of the selection test from Tajima (D) and Fu and Li (D*). However, a significant difference was observed between Malawi and other populations, as contrary to negative test values observed in other regions, suggesting an excess of low frequency polymorphisms (in line with high level of singleton haplotypes), Malawi exhibited positive and significant values (for D*) suggesting that this gene is under selection in this southern African populations in line with low haplotype diversity observed.

**Table 1:** Summary statistics for polymorphism at a fragment of the sodium channel gene in *An. funestus* populations from the four countries

Samples	N	S	π (k)	h(hd)	D	D*
Benin	20	10	0.0025(2.17)	10(0.89)	−0.79^ns^	−0.97^ns^
Uganda	38	19	0.0031(2.74)	19(0.89)	−1.30 ^ns^	−1.89 ^ns^
Kenya	32	21	0.0034(2.98)	19(0.95)	−1.47 ^ns^	−1.41 ^ns^
Malawi	20	9	0.004(3.5)	5(0.64)	1.36^ns^	1.38^*^
Total	110	35	0.0037(3.24)	41(0.94)	−1.6^ns^	−3.49^*^

N = number of sequences (2n); S, number of polymorphic sites; π, nucleotide diversity (k = mean number of nucleotide differences); h, number of haplotypes (hd = haplotype diversity); D and D* Tajima’s and Fu and Li’s statistics; ns, not significant

#### Population substructure at VGSC across Africa

Analysis of the genetic structure pattern at the VGSC fragment further supported the difference in genetic diversity observed between the Malawian populations and the West and East Africa populations. The construction of a maximum likelihood phylogenetic tree of *VGSC* sequences revealed that haplotypes from Malawi always cluster together separately from other countries (Fig. 5c). This difference is further supporter by the consistently high genetic differentiation estimates observed between Malawi and the other 3 countries (0.13 < K_ST_ < 0.17) whereas, the three West and East African populations exhibited low levels of genetic differentiation (0.035 < K_ST_ < 0.057) (Table 2) and cluster together on the Neighbor-joining tree of the genetic distances (Fig. 5d). This pattern of gene flow correlates with the genetic structure profiles of *An. funestus* populations across Africa also obtained with the DDT resistance gene GSTe2 [49] or with microsatellite markers [50, 51].

**Table 2:** Patterns of genetic differentiation between *An. funestus* population based on *K*_*ST*_ estimates from VGSC with (Nm)

	Malawi	Benin	Uganda
Benin	0.15***(0.72)		
Uganda	0.17***(0.59)	0.035**(3.1)	
Kenya	0.132***(0.81)	0.0057 ns (20.1)	0.04*(9.5)

PERMTEST calculates Hudson’s K_ST_ statistic of genetic differentiation. K_ST_ is equal to 12KS/KT, where KS is a weighted mean of K1 and K2 (mean number of differences between sequences in subpopulations 1 and 2, respectively) and KT represents the mean number of differences between two sequences regardless of their subpopulation. *, 0.01 < *P* < 0.05; **, 0.001 < *P* < 0.01; ***, *P* < 0.001

## Discussion

Elucidating mechanisms of insecticide resistance is crucial in understanding the evolution and dynamic of resistance in field populations of vectors and to help design suitable resistance management strategies. This study investigated the potential role of the knockdown resistance mechanism in the increasingly reported pyrethroid and DDT resistance in the major malaria vector *An. funestus* across Africa.

### Role of knockdown resistance is different to that observed in *An. gambiae s.l.*

This study has confirmed previous reports that the role of knockdown resistance in pyrethroid/DDT resistance in *An. funestus* is different from that observed in other major malaria vectors from *An. gambiae* complex, notably *An. gambiae s.s., An. coluzzii* and *An. arabiensis*. Indeed, despite an extensive effort to detect pyrethroids/DDT *kdr* mutations, the common L1014F mutation currently widespread in *An. gambiae s.s.* across Africa, was completely absent in *An. funestus* as previously observed [8, 12, 14, 18]. The absence of the 1014F mutation in *An. funestus* populations could also explain the lack of cross-resistance between pyrethroids and DDT in some regions such as in southern Africa where populations are highly resistant to pyrethroids but susceptible to DDT [5, 6] although moderate DDT resistance has recently been reported in this region [9]. This difference between *An. funestus* and *An. gambiae* is also observed for the other target-site resistance mechanisms in the acetylcholinesterase gene where the common G119S mutation conferring carbamates and organophosphates resistance in *An. gambiae* and other mosquitoes such as *Cx quinquefasciatus* (Weill et al. 2004) is completely absent in *An funestus* [5, 8] but rather the N485I mutation is found in *An. funestus* conferring bendiocarb resistance [52]. Furthermore, synergist assays performed with *An. funestus* across Africa (Riveron et al. 2014; Mulamba et al. 2014) also suggest that knockdown resistance is not a major mechanism contrary to what is observed in *An. gambiae* or *Cx quinquesfaciatus* and even *Ae. aegypti* highlighting the fact that one should not assume that mechanisms present in one *Anopheles* species apply to all. However, since most samples in this study are from 2009 and 2010, further studies should be performed with recent samples to assess whether the situation remains the same across Africa.

### Metabolic resistance remains the predominant pyrethroid resistance mechanism in *An. funestus* across Africa

The absence of the 1014F/S mutation and other confirmed *kdr* mutations in this study supports the predominant role that metabolic resistance plays in the pyrethroid resistance in *An. funestus* as shown previously [23, 25, 26]. This is in contrast to other mosquito species such as *An. gambiae* for which knockdown resistance plays a significant role [30]. However, despite the absence of the 1014F mutation in *An. funestus*, the detection of several amino acid replacements in the sodium channel gene in this species across Africa suggests that one cannot rule out a future role of knockdown resistance in pyrethroids and DDT resistance in this species. Indeed, the detection of the two amino acid changes L802S and T1008 M in Mozambique and that of I877L, V881 L and A1007S in Cameroon [18] or the F1021C in Uganda [12], albeit always at very low level suggests that such *kdr* resistance could occur in *An. funestus* but in other codons than 1014 as reported in *Aedes aegypti* with the mutations at the 1011, 1016 and 1534 codons [31, 32]. It is necessary to continue monitoring the frequency of the L802S and T1008 M and other mutations in field populations to assess their possible role in pyrethroid resistance. This is particularly important when taking into consideration the concept of a “tipping-point” in the evolution of a mutation in a population under selection as most *Anopheles* populations are currently with the scale up of insecticide-based control intervention throughout Africa. It is know that a resistance mutation can be present at low but gradually increasing frequency in a mosquito population for many years without being detected [1]. However, once the tipping-point is reached after continuous selection pressure, the frequency of that resistance mutation may shoot up rapidly as seen with the rapid spread of the 1014F mutation in *An. gambiae* population across Africa [30] or for the *kdr* mutation in *Ae. aegypti* populations in Mexico [1]. On the other hand, it is also possible that the observed amino acid changes have no association with knockdown resistance and just represent the natural polymorphism of the gene. Future monitoring will help establish their role.

### Genetic diversity of VGSC supports a limited role of knockdown resistance and suggests restriction to gene flow

The high genetic diversity observed for the VGSC fragment across Africa further supports that knockdown resistance currently plays no role in the pyrethroid/DDT resistance in *An. funestus*. This lack of selection is supported by the high number of substitutions and high haplotypes number observed throughout Africa with most of the haplotypes been singletons. The absence of selection on this gene in *An. funestus* is in marked contrast to the extensive selective sweep reported around the VGSC in *An. gambiae* with significant reduced genetic and haplotype diversity [53]. The restricted gene flow observed in this study between southern Africa and other regions is in line with previous analyses of the genetic diversity of different insecticide resistance genes such as the DDT resistance gene *GSTe2* [49] or the *CYP6P9a* and *CYP6P9b* pyrethroid resistance genes [22, 51, 54]. It is also in line with patterns of differentiation obtained with microsatellite markers [22, 50] further supporting a strong genetic differentiation between the southern African populations to those from East and West Africa. This further supports the presence of barriers to gene flow between *An. funestus* populations across Africa as previously suggested [22, 50]. Such restriction to gene flow could limit the spread of resistance mutations between populations of this species from different regions of Africa or lead to heterogeneous biological and genetic profile of this vectors across the continent.

## Conclusion

Investigation of knockdown resistance in the malaria vector *An. funestus* did not establish a major role for this resistance mechanism in the pyrethroid or DDT resistance in this species across Africa despite the detection of some non-synonymous mutations. Nevertheless, these mutations in the VGSC gene should be constantly monitored to detect any increased frequency that could be associated with selection of this resistance mechanisms in *An. funestus* populations.

## Additional files

Additional file 1: Table S1. Pyrosequencing primers information for the Voltage-Gated Sodium channel gene mutations. **Table S2.** List of primers used to sequence full VGSC. (DOCX 15 kb)

Additional file 2: Figure S1. Pyrosequencing genotyping of L1014 position in An. funestus. A) is the chromatogram showing the wild TTA genotype for L1014 whereas B) is the heterozygote TTA/T for L1014/1014F observed at 2 mosquitoes but not confirmed by sequencing. (TIFF 33 kb)
