# Supplementary material for: Investigating knockdown resistance (kdr) mechanism against pyrethroids/DDT in the malaria vector Anopheles funestus across Africa
Source: BMC Genet. 2017 Aug 9;18:76. doi: 10.1186/s12863-017-0539-x (PMC5549319; doi:10.1186/s12863-017-0539-x)
Supplement: Supplementary file 1 — Pyrosequencing primers information for the Voltage-Gated Sodium channel gene mutations. Table S2. List of primers used to sequence full VGSC. (DOCX 15 kb) [file 12863_2017_539_MOESM1_ESM.docx]

**Table S1:** Pyrosequencing primers information for the Voltage-Gated Sodium channel gene mutations.

|  | ***L1014F*/S** | **L802S** | **T1008M** | **L928R** | **F938W** | **L913F** | **L932F** |
| --- | --- | --- | --- | --- | --- | --- | --- |
| **Forward primer** | TTGTGTTCCGTGTGCTATGC | CGACCCATTTGTGGAGCTAT | TTGTGTTCCGTGTGCTATGC | AACGATGGGAGCGTTAGGTA | AACGATGGGAGCGTTAGGTA | TCTTTAAGCTCGCTAAATCGTG | TCTTTAAGCTCGCTAAATCGTG |
| **Biotinalated Reverse primer** | AAAAACGATCTTGGTCCATGT | AAGCTTCATTGTTGCTTCGAT | ATCGGTGAACACTCACGACT | TCCGCTGTGATTTATGGTTG | TCCGCTGTGATTTATGGTTG | TCCGCTGTGATTTATGGTTG | TCCGCTGTGATTTATGGTTG |
| **Sequencing** | TGTAGTGATAGGAAAT | TACGTTATTCATGGCGT | ACCATTTTTCCTGGCT | GGGAGCGTTAGGTAAT | GGGAGCGTTAGGTAAT | CTGGCCAACGCTGAAT | GYAATYTGACGTTCGT |
| **Sequence to analyse** | T C/T A/T GTCGTAAG | C/T AGATCATCATG | A C/T GGTAGTAATAG | C G/T GACGTTCGTGCTCTGCATTATCATCTTCAG/TCTG/TG/TGC | CG/TGACGTTCGTGCTCTGCATTATCATCTTCAG/TCT G/T G/TGC | C/T T A/T CTCATTTCCATCA | GC/T T A/C TGCATYATCA |
| **Product size (bp)** | 154 | 162 | 154 | 177 | 177 | 156 | 156 |
| **Allele** | C/T//A/T | C/T | C/T | G/T | G/T | C/T | C/T |

**Table S2: List of primers used to sequence full VGSC**

| **Names** | **Primer sequence** |
| --- | --- |
| KdrfullF | ATG ACC GAA GAC TCC GAT TCG AT |
| KdrfullR | TAA CAT CGC GAA CGG CAG ATG TCT GA |
| Exon6kdrF | CATCGTCGGAGCTGTCATAG |
| Exon18kdrF | GGCAAAATCATGGCCTACAT |
| Exon27kdrF | TTCGTTGCTTCACTTTGTGG |
| Exon27kdrR | CACGTTGAAGATGGACGGTA |
| Exon13kdrF | CGCTGAAACAAACCATAAAGG |
| Exon16kdrR | TCTGTGGGGAAGTAATAAACAGAG |
| Exon19kdrR | GCA TAG CAA AAG CAC CCT TCA GC |
| KdrEx19F | TTT TTA AGC TCG CTA AAT CGT G |
| KdrEx19R | CAG TAA CCA GCG GAC ATT GA |
| KdrEx20R | CCG AAA TTT GAC AAA AGC AAA |
