# Supplementary figures and images for: Investigating knockdown resistance (kdr) mechanism against pyrethroids/DDT in the malaria vector Anopheles funestus across Africa
Source: BMC Genet. 2017 Aug 9;18:76. doi: 10.1186/s12863-017-0539-x (PMC5549319; doi:10.1186/s12863-017-0539-x)

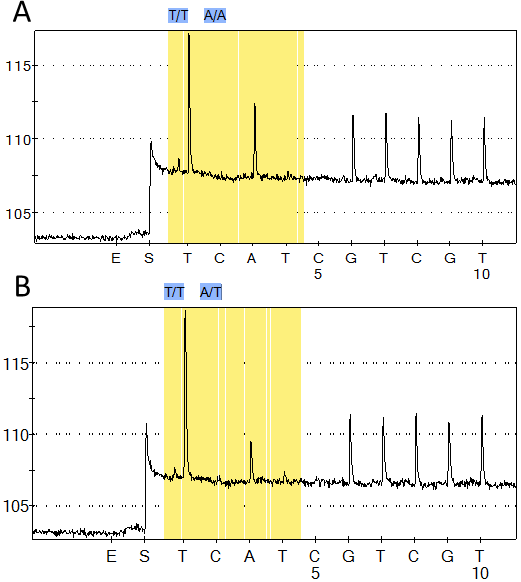

Supplement: Supplementary file 2 — Pyrosequencing genotyping of L1014 position in An. funestus. A) is the chromatogram showing the wild TTA genotype for L1014 whereas B) is the heterozygote TTA/T for L1014/1014F observed at 2 mosquitoes but not confirmed by sequencing. (TIFF 33 kb) [file 12863_2017_539_MOESM2_ESM.tif]
